# Spatial heterogeneity of FGFR2b in gastric cancer: a comparative analysis of primary tumors and peritoneal dissemination

**DOI:** 10.1007/s00428-025-04233-z

**Published:** 2025-08-30

**Authors:** Haruki Ogawa, Hiroyuki Abe, Koichi Yagi, Yoshifumi Baba, Yasuyuki Seto, Tetsuo Ushiku

**Affiliations:** 1 Department of Pathology, Graduate School of Medicine, the University of Tokyo, 7-3-1 Hongo, Bunkyo-Ku, 113-0033 Tokyo Japan; 2 Department of Gastrointestinal Surgery, Graduate School of Medicine, the University of Tokyo, 7-3-1 Hongo, Bunkyo-Ku, 113-0033 Tokyo Japan; 3 Department of Esophageal/Gastric Surgery, National Cancer Center Hospital, 5-1-1 Tsukiji, Chuo-Ku, 104-0045 Tokyo Japan

**Keywords:** Gastric cancer, FGFR2b, Peritoneal dissemination, Molecular targeted therapy, Biomarker testing

## Abstract

Gastric cancer with peritoneal dissemination (PD) confers poor prognosis and limited treatment options. FGFR2b-targeted therapy has emerged as a potential approach for FGFR2b-positive tumors. However, the expression and amplification status of FGFR2b in PD remains poorly characterized. This study aimed to investigate FGFR2b expression and gene amplification in matched primary tumors and PD tissues from gastric cancer patients, and to evaluate their association with established biomarkers including HER2, CLDN18, and PD-L1. Immunohistochemistry (IHC) for FGFR2b was performed on matched primary and PD tissues from 84 patients. *FGFR2* FISH was conducted in IHC-positive cases. FGFR2b expression was detected in 7.1% (6/84) of primary tumors and 4.8% (4/84) of PD samples. Expression was highly heterogeneous; only one case was FGFR2b-positive in both primary and PD tissues. *FGFR2* amplification was found in 6 of 10 IHC-positive samples, and not observed in IHC-negative samples. FGFR2b status showed no significant correlation with HER2, CLDN18, or PD-L1. FGFR2b expression in gastric cancer is spatially heterogeneous and discordant between primary tumors and PD. It may be preferable to test both primary tumor and PD tissue, if available, to better identify candidates for FGFR2b-targeted therapy. FGFR2b represents a potential therapeutic target for a subset of PD-positive gastric cancers lacking other biomarker expression.

**Supplementary Information:**

The online version contains supplementary material available at 10.1007/s00428-025-04233-z.

## Introduction

Gastric cancer is the fifth leading cause of cancer-related mortality worldwide [1]. The prognosis of advanced-stage disease, particularly in patients with peritoneal dissemination (PD), remains extremely poor [2]. Standard therapies for unresectable or recurrent gastric cancer include chemotherapy, anti-HER2 therapy (e.g., trastuzumab), anti-VEGFR2 therapy (e.g., ramucirumab), and immune checkpoint inhibitors. More recently, zolbetuximab, an anti-claudin 18.2 antibody, has been added to the therapeutic option. PD-positive gastric cancers typically exhibit a diffuse-type histology and this subtype is usually HER2-negative. PD-L1expression is also relatively low in this subtype, when compared to the microsatellite instable and Epstein-Barr virus-positive subtypes [3]. In our previous study, CLDN18-positivity in PD-positive gastric cancer was observed in only 28% of primary tumors and 20% of PD tissues [4], underscoring the need for alternative therapeutic targets.

Bemarituzumab, a monoclonal antibody against the fibroblast growth factor receptor 2b (FGFR2b), has shown promising efficacy in a randomized phase 2 trial (FIGHT, NCT03694522) in combination with chemotherapy for HER2-negative, FGFR2b-positive advanced gastric cancer [5]. FGFR2b positivity was defined in the final analysis of FIGHT study as ≥ 10% of tumor cells showing moderate or strong membranous staining. This definition has been carried forward in the ongoing phase 3 FORTITUDE trials (FORTITUDE-101, NCT05052801 and FORTITUDE-102, NCT05111626).

Although a few reports have described FGFR2b expression patterns comparing primary and metastatic tumors [6–8], no study has focused on FGFR2b expression in PD. Because of its clinical importance and the lack of data, we examined the expression of FGFR2b in primary tumors, PD tissues, and lymph node metastases from gastric cancer patients with PD. *FGFR2* amplification for FGFR2b-positive cases was also assessed using fluorescence in situ hybridization (FISH). In addition, we analyzed the association of FGFR2b status with other biomarkers, including HER2, PD-L1 and CLDN18.

## Materials and methods

### Tissue samples

This retrospective study included 84 gastric cancer cases with matched tissue samples from primary tumors and peritoneal dissemination (PD), collected from the pathology archive of The University of Tokyo Hospital between April 2000 and December 2021. This cohort was previously analyzed in our CLDN18 study [4]. Primary tumor samples included 78 biopsies, 16 surgical specimens, and six autopsy cases. PD samples comprised 78 surgical and six autopsy specimens. Additionally, 28 lymph node metastasis specimens were analyzed. Surgically resected and autopsy specimens were collected from chemotherapy-naïve patients to evaluate intratumoral heterogeneity prior to chemotherapy, whereas the remaining specimens were collected regardless of chemotherapy history.

Clinicopathological variables including age, sex, tumor location, tumor size, T stage, lymphatic invasion, and venous invasion were retrieved from pathology records. Tumor histology was classified according to the Lauren classification [9].

Follow-up data were obtained from medical records. Four patients in whom gastric cancer was first diagnosed at autopsy were excluded from survival analyses. Overall survival (OS) was defined as the time from diagnosis to death from any cause or last follow-up. Recurrence-free survival was not analyzed, as patients with PD typically did not undergo curative resection.

This study was conducted in accordance with the Declaration of Helsinki and was approved by the institutional review board (approval number 10461–13).

### Immunohistochemistry

Formalin-fixed paraffin-embedded (FFPE) tissues were sectioned at 3 µm thickness. Immunohistochemistry (IHC) was performed using a Ventana Benchmark Ultra automated stainer (Ventana Medical Systems, Tucson, AZ, USA), following the manufacturer's protocol. FGFR2b IHC was conducted using a mouse-rat chimeric monoclonal antibody (clone mFR2-10b; Daiichi Sankyo Co., Ltd., Japan), since the FPR2-D clone used in the FIGHT trial was not available at the time of study.

FGFR2b membranous staining was scored for intensity (0 = none, 1 +  = weak, 2 +  = moderate, 3 +  = strong) and percentage of positive tumor cells. The intensity was evaluated in accordance with a previously published study employing the same antibody (mFR2-10b) [6]. Gastric cancer tissue with confirmed *FGFR2* amplification and strong expression (3 +) was used as a 3 + positive control. FGFR2b positivity was defined as moderate (2 +) or strong (3 +) membranous staining in ≥ 10% of tumor cells, consistent with the eligibility criteria of the ongoing phase 3 trial.

Two independent observers (H.O. and H.A.), blinded to clinical data, evaluated all samples. In cases of disagreement, slides were re-reviewed jointly to reach consensus.

Data for HER2, PD-L1, and CLDN18 expression were derived from our previous study on the same cohort [4].

HER2: IHC was performed using a rabbit monoclonal anti-HER2 antibody (clone 4B5, prediluted; Roche). Scoring followed the ASCO/CAP guidelines [10]. Equivocal (2 +) cases were further tested for amplification by dual-color in situ hybridization (DISH) using the INFORM HER2 Dual ISH Kit (Roche). HER2-positive status was defined as IHC 3 + or IHC 2 + with gene amplification.

PD-L1: IHC was performed with clone SP263 (prediluted; Roche). PD-L1 expression was quantified using the combined positive score (CPS), defined as the number of PD-L1–positive tumor cells, lymphocytes, and macrophages divided by total tumor cells and multiplied by 100 [11]. Expression levels were categorized as CPS < 1, 1 ≤ CPS < 5, and CPS ≥ 5, based on CheckMate 649 trial criteria [12].

CLDN18: IHC was performed using clone 43-14A (Ventana). Membranous staining intensity and proportion were assessed. CLDN18 positivity was defined as moderate (2 +) or strong (3 +) staining in ≥ 75% of tumor cells, based on eligibility criteria from the SPOTLIGHT and GLOW trials [13, 14].

### Fluorescence in situ hybridization (FISH) of FGFR2

FISH was performed on 4 µm FFPE sections to assess *FGFR2* gene amplification, but only in cases with FGFR2b expression in either the primary tumor or PD, given the low likelihood of amplification in FGFR2b-negative tumors [15]. Both primary and PD tissues were examined per case.

We used the *FGFR2*/*CEN10p* Dual Color Probe (GSP Lab, Inc., Hyogo, Japan). *FGFR2* was labelled red, and *centromere* *10* (*CEN10*) green. After probe hybridization, ≥ 50 tumor nuclei were assessed per sample. The *FGFR2*/*CEN10* ratio was calculated, and a ratio > 2.0 was considered *FGFR2* amplification positive.

### Statistical analysis

All analyses were conducted using JMP Pro 17 (SAS Institute Inc., Cary, NC, USA). Continuous variables were compared using the paired Student’s t-test, and categorical variables using Fisher’s exact test. OS was estimated by the Kaplan–Meier method and compared using the Wilcoxon test. Hazard ratios (HRs) and 95% confidence intervals (CIs) were calculated using the Cox proportional hazards model. A *p*-value < 0.05 was considered statistically significant.

## Results

### FGFR2b status in primary tumor and its clinicopathological correlation

**Fig. 1:**
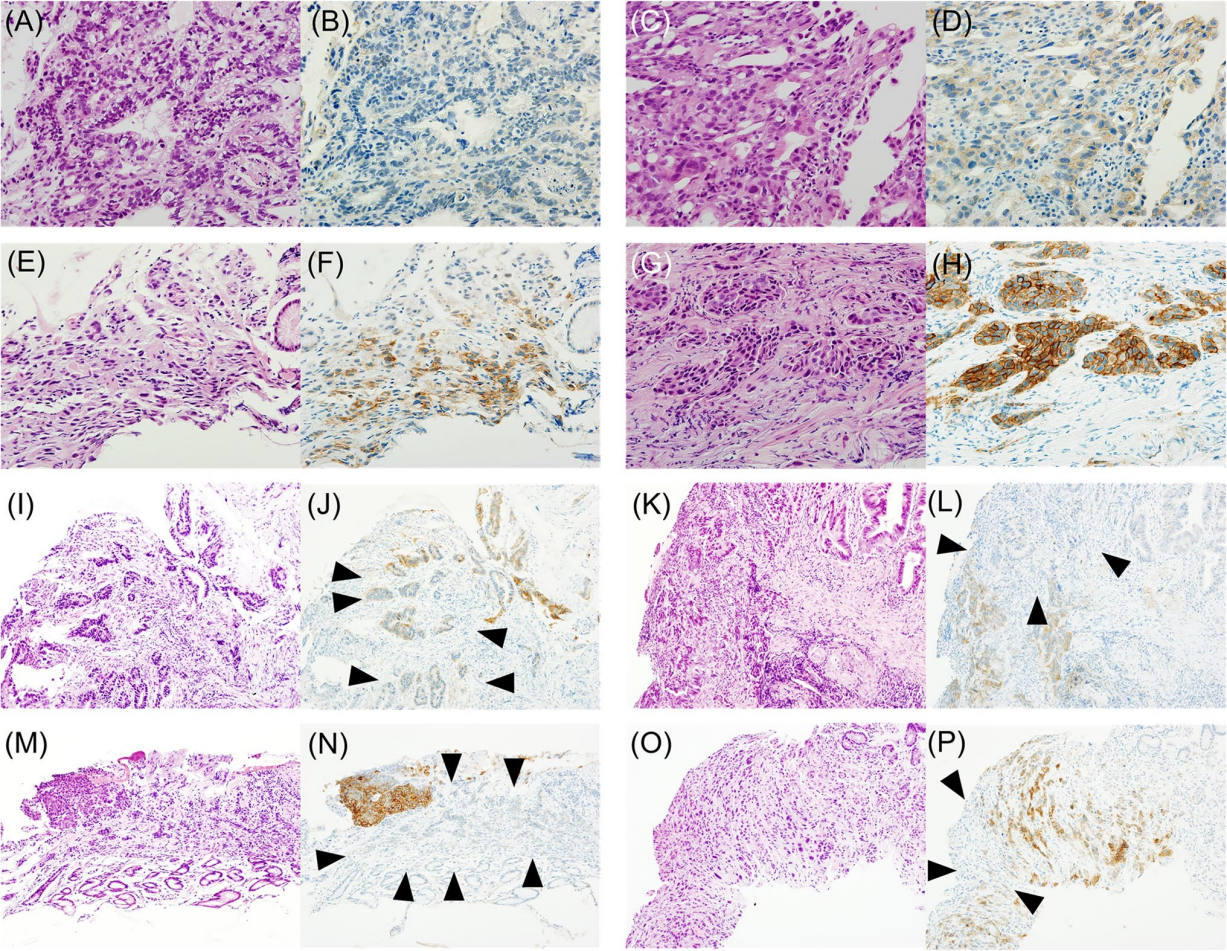
Representative FGFR2b immunohistochemistry images. Paired H&E and FGFR2b immunohistochemistry images illustrating four scoring categories of FGFR2b expression: 0, none (**A**, **B**); 1 +, weak (**C**, **D**); 2 +, moderate (**E**, **F**); and 3 +, strong (**G**, **H**). Panels I–P show additional paired images (H&E: **I**, **K**, **M**, **O**; FGFR2b: **J**, **L**, **N**, **P**) from four cases demonstrating intratumoral heterogeneity in FGFR2b expression. Arrowheads indicate tumor regions lacking FGFR2b expression

Representative immunohistochemistry images showing each FGFR2b intensity score are presented in Fig. 1A–H. Among the 84 primary gastric tumors, FGFR2b expression was positive in six cases (7.1%) (Table 1). No significant associations were observed between FGFR2b status and sex, age, or tumor location. Histologically, 66 cases (78.6%) were of the diffuse type and 18 (21.4%) of the intestinal type. FGFR2b expression did not differ significantly between histological subtypes.

**Table 1:** FGFR2b status and clinicopathological characteristics in primary tumors and PD

		FGFR2b in primary tumors			FGFR2b in PD		
	n	−	+	*P*-value^*^	−	+	*P*-value^*^
Total	84	78	6		80	4	
Sex				0.17			0.46
Male	55	49	6		51	4	
Female	29	29	0		29	0	
Age				0.23			0.14
<65	44	41	3		41	3	
≥65	40	37	3		39	1	
Tumor site				0.52			0.39
U	17	15	2		17	0	
M	51	48	3		47	4	
L	16	15	1		16	0	
Histologic type				0.54			0.49
intestinal	18	16	2		17	1	
diffuse	66	62	4		63	3	
FGFR2b in primary tumors							0.80
negative		N.A.			75	3	
positive					5	1	

*PD* peritoneal dissemination, *N.A.* not applicable

*Fisher’s exact test

**Fig. 2:**
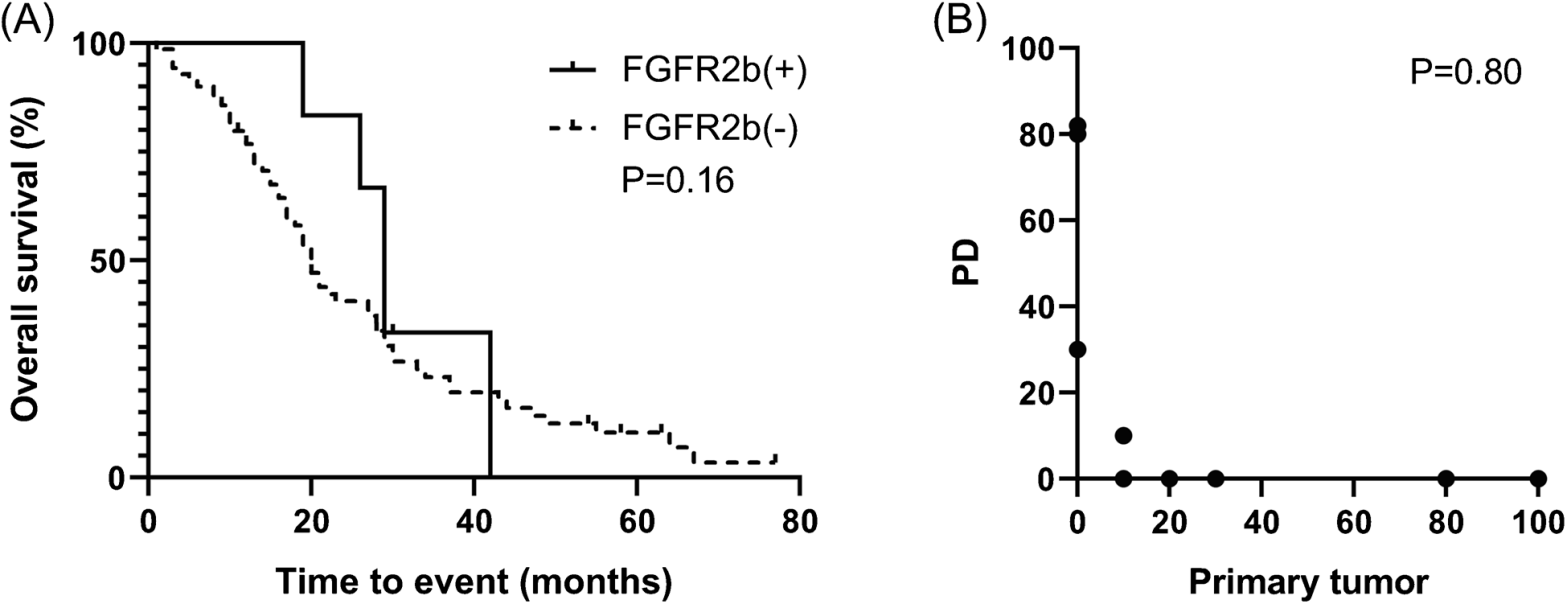
FGFR2b status in the primary tumors and its association with patient survival and peritoneal dissemination. **A** Kaplan–Meier survival analysis comparing FGFR2b-positive and FGFR2b-negative groups revealed no significant difference in overall survival. **B** Scatter plot showing the percentage of FGFR2b-positive tumor cells in the primary tumor (X-axis) versus the extent of peritoneal dissemination (PD) (Y-axis). Only one case showed FGFR2b positivity in both the primary tumor and PD, with 10% of tumor cells positive in each site. PD, peritoneal dissemination

Kaplan–Meier analysis revealed no significant difference in overall survival (OS) between patients with FGFR2b-positive and FGFR2b-negative tumors (Fig. 2A).

Intratumoral heterogeneity of FGFR2b expression was evident in 4 of the 6 positive biopsy cases (Fig. 1I–P). Only one surgically resected primary tumor was FGFR2b-positive. Although the tumor showed full-thickness invasion of the gastric wall, FGFR2b expression was limited to approximately 10% of the cancer cells located in the submucosal layer at the tumor periphery.

### Comparison of FGFR2b expression between primary tumor, PD, and lymph node metastasis

FGFR2b expression in PD samples was observed in four of 84 cases (4.8%) (Fig. 2B and Table 1). No significant correlation with age, sex, or tumor location was identified. Among the nine cases positive for FGFR2b in either the primary or PD tumor, only one case (1.2%) showed FGFR2b positivity in both sites.

Of the six cases with FGFR2b-positive primary tumors, only one (16.7%) also showed FGFR2b expression in PD. In contrast, 75 of 78 cases (96.2%) with FGFR2b-negative primary tumors were also negative in PD.

FGFR2b expression in lymph node metastases was observed in 6 of 28 cases (21.4%). All three cases with FGFR2b-positive primary tumors also showed FGFR2b-positive in lymph node metastases. In contrast, 22 of 25 cases (88%) with FGFR2b-negative primary tumors were also FGFR2b-negative in lymph node metastases.

### *FGFR2* gene amplification and its correlation with FGFR2b expression

**Fig. 3:**
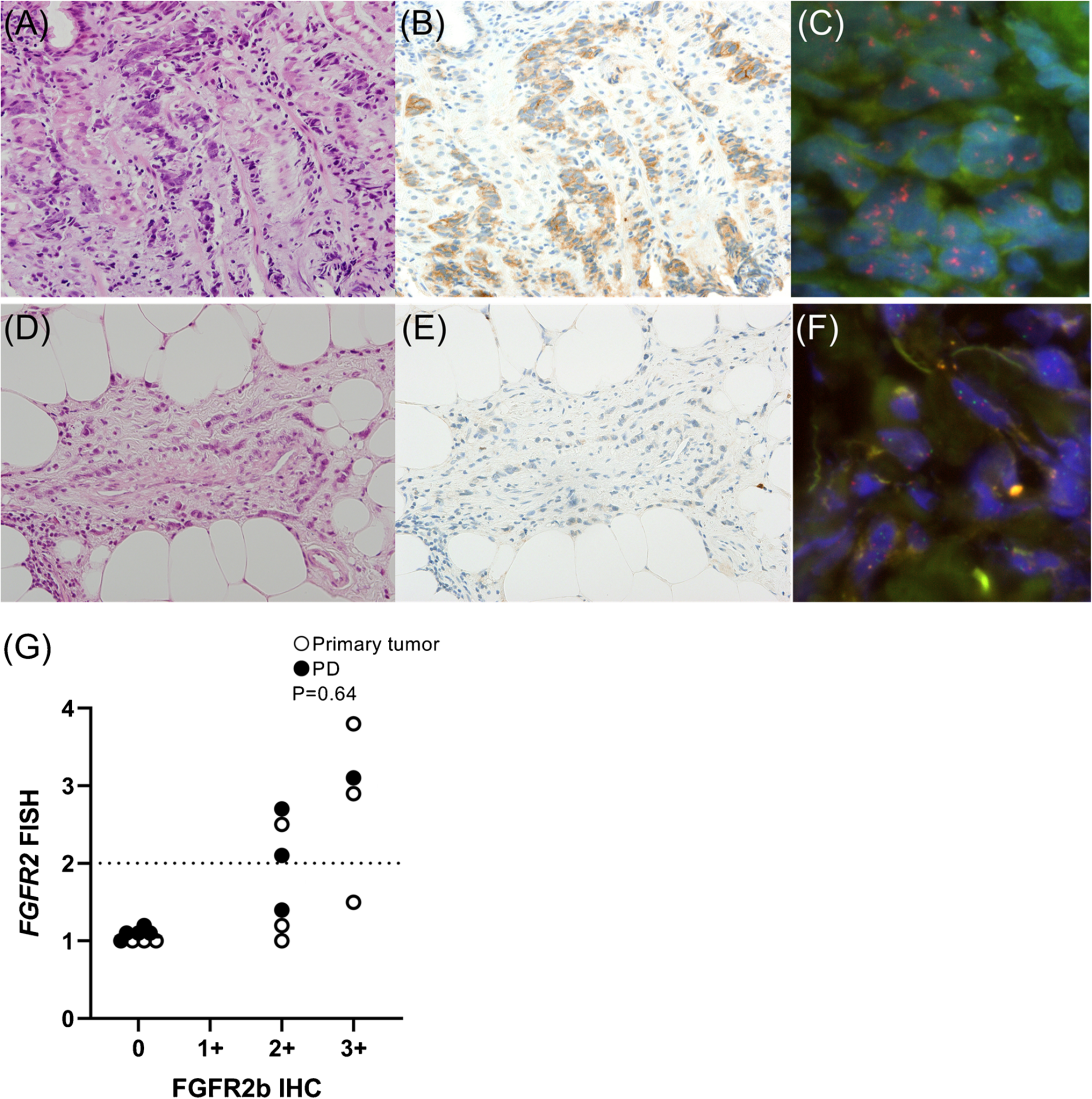
FGFR2b expression and *FGFR2* gene amplification by FISH. Paired images of a primary tumor (**A**–**C**) and matched peritoneal dissemination (PD) sample (**D**–**F**): H&E staining (**A**, **D**); FGFR2b immunohistochemistry (**B**, **E**); *FGFR2* FISH (**C**, **F**). In the primary tumor, FGFR2b expression was strong (3 +) in 80% of tumor cells (**B**), with *FGFR2* gene amplification confirmed by FISH (*FGFR2*/*CEN10* ratio = 2.93, **C**). In the PD lesion, FGFR2b expression was negative (**E**), and no *FGFR2* amplification was detected (*FGFR2*/*CEN10* ratio = 1.05, **F**). Red signals indicate *FGFR2*; green signals indicate *CEN10*. (**G**) Correlation between FGFR2b IHC score (X-axis) and *FGFR2* amplification by FISH (Y-axis, *FGFR2*/*CEN10* ratio). All IHC-negative cases lacked gene amplification, whereas 6 of 10 cases (60%) cases with 2 + or 3 + IHC demonstrated *FGFR2* amplification. IHC, immunohistochemistry

Representative images of FGFR2b immunohistochemistry and *FGFR2* FISH are shown in Fig. 3A–F. Among nine cases with FGFR2b expression in either the primary tumor or PD, 10 tissue specimens (6 primary tumors and 4 PD samples) were FGFR2b-positive and 8 were negative (3 primary tumors and 5 PD samples). FISH was performed on all 18 specimens (Fig. 3G and Supplementary table).

None of the FGFR2b-negative specimens showed *FGFR2* amplification. Among FGFR2b-positive specimens, *FGFR2* amplification was detected in 6 of 10 cases (60%). Specifically, amplification was observed in 3 of 6 cases (50%) with 2 + staining and in 3 of 4 cases (75%) with 3 + staining.

In tumors with intratumoral heterogeneity, *FGFR2* amplification was restricted to FGFR2b-positive regions. All six tumors with ≥ 30% FGFR2b-positive cells demonstrated gene amplification, while none of the three cases with < 30% positivity was amplified.

### Correlation between FGFR2b and other biomarkers (CLDN18, HER2, and PD-L1)

**Fig. 4:**
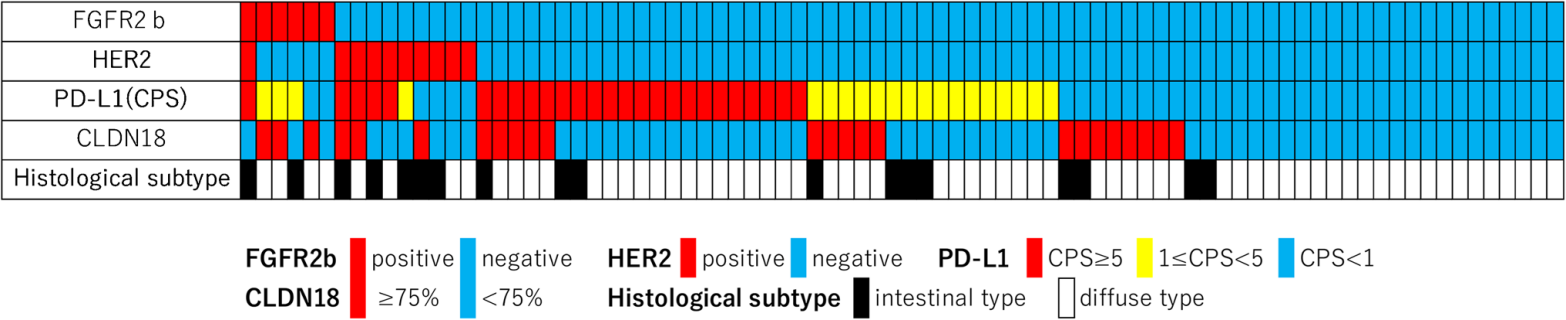
Expression profiles of FGFR2b, HER2, PD-L1, and CLDN18. Comparison of FGFR2b expression status with HER2, PD-L1 (combined positive score [CPS]), and CLDN18 expression in each case. No significant correlations were observed between FGFR2b and any of these biomarkers

FGFR2b expression data were integrated with previously reported HER2, PD-L1, and CLDN18 results in the same cohort [4] (Fig. 4). HER2 positivity was observed in 10 of 84 cases (11.9%), including 6 of 18 (33.3%) intestinal-type tumors and 4 of 66 (6.1%) diffuse-type tumors. PD-L1 was CPS ≥ 5 in 26 cases (31.0%), 1 ≤ CPS < 5 in 20 cases (23.8%), and CPS < 1 in 38 cases (45.2%). CLDN18 positivity (≥ 75%) was seen in 24 of 84 cases (28.6%).

No significant correlations were found between FGFR2b and HER2, PD-L1, or CLDN18 expression. Overall, 60 of 84 tumors (71.4%) were positive for at least one of these therapeutic targets. One case was positive for FGFR2b only, with no positive results for HER2, PD-L1, or CLDN18.

## Discussion

This study demonstrated that FGFR2b positive rates in PD-positive gastric cancers were 7.1% in primary tumors and 4.8% in PD lesions. These rates are lower than those reported in the FIGHT trial, where FGFR2b positivity was observed in 18% of primary tumors [16]. However, a recent large-scale study by Lee et al. reported FGFR2b overexpression in 4.1% of gastric cancer cases, using the same antibody clone (FPR2-D) and cut-off criteria as the FIGHT trial [8]. Therefore, differences in FGFR2b positivity rates are not necessarily solely due to differences in the antibodies used, but may also be influenced by cohort differences.

Our study is the first to evaluate FGFR2b expression with a special focus on PD lesions. Only one case showed FGFR2b positivity in both the primary tumor and PD, indicating a low concordance rate. Similarly, Lee et al. reported concordance in only one (0.7%) of 135 paired primary and metastatic tumor specimens [8]. Interestingly, FGFR2b status was concordant between primary tumors and lymph node metastases in 25 of 28 cases (89%), suggesting that FGFR2b expression may be better preserved in lymph node metastases than in PD. These findings suggest that FGFR2b overexpression may emerge in subclonal tumor populations and be differentially maintained across metastatic sites, contributing to spatial heterogeneity.

Several mechanisms may explain the discordance between primary tumors and PD lesions. FGFR2b-positive clones may fail to metastasize, or alternatively, amplification and overexpression may be acquired during dissemination. Notably, some PD lesions were FGFR2b-positive despite being derived from FGFR2b-negative primary tumors, highlighting the importance of assessing PD tissue directly. In line with this, Lee et al. also observed low sensitivity of biopsy specimens in detecting FGFR2b expression, with only one biopsy-surgical pair (0.6%) showing concordance [8]. These observations suggest the clinical importance of testing both primary and metastatic lesions when considering FGFR2b-targeted therapies such as bemarituzumab.

We also examined the relationship between FGFR2b expression and *FGFR2* gene amplification. *FGFR2* amplification was present in 60% of FGFR2b IHC-positive tumor specimens and absent in all IHC-negative ones. Consistent with our findings, an earlier study reported amplification in 94.1% of FGFR2b-overexpressing tumors, and none among FGFR2b-negative cases [8]. Previous publications have reported that the positive rate of FGFR2b expression was 4–28%, while the *FGFR2* amplification rate was 2–9%, which is equal to or lower than the positive rate of FGFR2b expression [5, 6, 15, 17–22]. These results suggest that FGFR2b IHC is a reliable surrogate marker for amplification and is sufficient to select gastric cancer patients for treatment with FGFR2 inhibitors.

Notably, we observed no significant correlation between FGFR2b expression and other biomarkers such as HER2, CLDN18, or PD-L1. This is consistent with previous studies, showing no significant overlap between FGFR2b and other actionable biomarkers [8, 23]. Importantly, one case in our study was positive only for FGFR2b, suggesting that it may represent an independent therapeutic niche within biomarker-negative gastric cancer.

This study has limitations. It was retrospective and based on a single institution. The number of cases was relatively small because PD is infrequently surgically resected in clinical practice. Additionally, in the present study, FGFR2b status in most primary tumors was assessed using biopsy specimens. Due to the small sample size and the intratumoral heterogeneity of FGFR2b expression, the positivity rate in primary tumors may have been underestimated. Second, because clone FPR2-D used in clinical trial was not available when we started this study, we used clone mFR2-10b antibody. The use of a non-FIGHT trial antibody may have influenced positivity rates, potentially limiting comparability with other studies. However, although no study has directly compared FPR2-D and mFR2-10b, comparable FGFR2b-positive ratios were reported in a study using FIGHT trial antibody [8]. Additionally, due to the low overall frequency of FGFR2b-positive cases, our survival analysis and other clinicopathological analyses were underpowered. Nevertheless, our study uniquely examines FGFR2b status in PD, highlighting the critical role of spatial heterogeneity.

In conclusion, to the best of our knowledge, this is the first study to evaluate FGFR2b status in a series of PD-positive gastric cancers, with a particular focus on comparing primary tumors and PD tissues. Given the spatial heterogeneity and low concordance of FGFR2b expression between primary tumors and PDs, assessing FGFR2b status in both sites may improve selection of candidates for FGFR2b-targeted therapy.

## Supplementary Information

Below is the link to the electronic supplementary material.

Supplementary file 1 (DOCX 18.2 KB)
