# Supplementary material for: Spatial heterogeneity of FGFR2b in gastric cancer: a comparative analysis of primary tumors and peritoneal dissemination
Source: Virchows Arch. 2025 Aug 30;488(6):1275–82. doi: 10.1007/s00428-025-04233-z (PMC13264584; doi:10.1007/s00428-025-04233-z)
Supplement: Supplementary file 1 — (DOCX 18.2 KB) [file 428_2025_4233_MOESM1_ESM.docx]

Supplementary table. FGFR2b immunohistochemistry and *FGFR2* FISH

| Case # |  | FGFR2b expression | FGFR2b intensity score | *FGFR2*/*CEN10* |
| --- | --- | --- | --- | --- |
| 1 | biopsy | 20% | 3+ | 1.54 |
|  | PD | 0% | 0 | 1.20 |
| 2 | biopsy | 0% | 0 | 1.13 |
|  | PD | 80% | 3+ | **3.13** |
| 3 | biopsy | 10% | 2+ | 1.42 |
|  | PD | 0% | 0 | 1.13 |
| 4 | biopsy | 80% | 3+ | **2.93** |
|  | PD | 0% | 0 | 1.05 |
| 5 | biopsy | 10% | 2+ | 1.23 |
|  | PD | 10% | 2+ | 0.95 |
| 6 | biopsy | 100% | 3+ | **3.82** |
|  | PD | 0% | 0 | 1.01 |
| 7 | biopsy | 0% | 0 | 1.10 |
|  | PD | 80% | 2+ | **2.71** |
| 8 | biopsy | 0% | 0 | 0.96 |
|  | PD | 30% | 2+ (positive area) | **2.09** |
|  |  |  | 0 (negative area) | 1.04 |
| 9 | biopsy | 30% | 2+ | **2.56** |
|  | PD | 0% | 0 | 0.99 |

PD, peritoneal dissemination.

*FGFR2*/*CEN10* ratios larger than 2 are in bold.
